# COST Action “EuroTelepath”: digital pathology integration in electronic health record, including primary care centres

**DOI:** 10.1186/1746-1596-6-S1-S6

**Published:** 2011-03-30

**Authors:** Marcial García Rojo, Ana Morillo Castro, Luis Gonçalves

**Affiliations:** 1 Pathology Department, Hospital General de Ciudad Real, Ciudad Real, Spain; 2 Community Health Center of Membrilla, Coidad Real, Spain; 3 Pathology Department, Hospital do Espiritu Santo, Évora, Portugal

## Abstract

**Introduction:**

Digital pathology includes the information technology that allows for the management of information, including data and images, generated in an anatomic pathology department.

**COST Action IC0604:**

The integration of digital slides in the electronic health record is one of the main objectives of COST Action IC0604 “Telepathology Network in Europe” (EURO-TELEPATH). Fostering use of medical informatics standards and adapting them to current needs is needed to manage efficiently extremely large medical images, like digital slide files.

**Digital slides in Pathology:**

Digital slides can play a role in disease prevention, primary diagnosis, and second opinion. In all these tasks, automated image analysis can also be a most valuable tool.

**Interoperability in pathology information systems:**

In order to achieve an efficient interoperability between pathology information systems with other clinical information systems, obtaining a seamless integration of pathology images (gross pictures and digital slides) with LIS-Pathology Information system in a web environment is an important task. Primary care information systems should also be included in the integration, since primary care centres play an essential role in the generation of clinical information and specimen collection. A common terminology, based in SNOMED CT is also needed.

**Conclusions:**

Main barrier in the integration of digital slides in pathology workflow and eHealth record is the cost of current digital slide scanners. Pathology information system vendors should participate in standardization bodies.

## Introduction

The concept digital pathology comprises the information technology that allows for the management of information, including data and images, generated in an anatomic pathology department. Anatomic pathology information system and digital imaging modalities are two main components of digital pathology.

Digital imaging solutions include image acquisition modalities, post processing stations, image viewers and picture image and distribution systems (PACS).

There are different types of digital image acquisition modalities in anatomic pathology, from macroscopic or microscopic still images to whole slide scanning images.

Photographic still images capture relevant information about specimen at a macroscopic level (photographs of specimen or derived specimen like paraffin blocks, etc). The acquisition modality for macroscopic (or gross) imaging is photo camera.

Microscopic images capture relevant information about specimen at a microscopic level. The main objective of virtual microscopy system or whole slide imaging (WSI) devices is building digital slides. They are capable of digitizing complete slides at high magnifications. Two main groups of acquisition modalities for microscopic images (WSI devices) can be distinguished: modalities using both a motorized microscope and a camera and slide scanners (table 1).

**Table 1:** Digital slide scanners in Pathology

System	Vendor	Objective/NA	# slides	Light path	File format
ACIS® III	Dako http://www.dako.com/	Nikon 4x, 10x, 20x, 40x, 60x	100	Halogen lamp	JPEG, TIFF, BMP
Applied Imaging Ariol®	Genetix http://www.genetix.com/	Motorized microscope. 1.25x, 5x, 10x, 20x (0.36 μm/pixel), 40x (0.18 μm/pixel)	50	Fluorescence available	JPEG
dotSlide™	Olympus http://www.olympus.co.uk/microscopy/22.htm	Motorized microscope. 2x plapon, 10x, 20x, 40x uplsapo	1 (50)	Halogen lamp. Fluorescence, polarisation available	Olympus VSI , JPEG, TIFF , JPEG2000
GoodSpeed®	Alphelys http://www.alphelys.com/	Motorized microscope	4 or 8	Dark field and fluorescence available	JPEG
iSan 2.0™	BioImagene http://www.bioimagene.com/	Olympus 20x/0.50 (0.46um/pixel) Plan Fluor Olypus 40x/0.75 (0.23um/pixel) Plan Fluor	160	Bright-field, integrated LED	JPEG2000, BIF, TIFF
iScan Coreo Au™	BioImagene http://www.bioimagene.com/	Automated turret with 4 objectives: Olympus 4X (0.1 NA), 10X (0.3 NA), 20X (0.5 NA), and 40X (0.75 NA)	160	Bright-field, integrated LED	JPEG2000, BIF, TIFF
iScan Concerto™	BioImagene http://www.bioimagene.com/	Automated turret with 4 objectives: Olympus 4X (0.13 NA), 10X (0.3 NA), 20X (0.5 NA), and 40X (0.75 NA) or 60X (0.90NA, 0.15 μm/pixel)	80	Integrated LED. Fluorescence available	JPEG2000, BIF, TIFF
Pannoramic Desk®	3DHistech http://www.3dhistech.com/	20x/0.80 (0.23 μm) Plan Apo. 40x/0.95 Plan Apo	1	Bright-field, halogen lamp	Mirax format, JPEG, TIFF
Pannoramic Midi®	3DHistech http://www.3dhistech.com/	20x/0.80 (0.23 μm) Plan Apo. 40x/0.95 Plan Apo	12	Fluorescence available	Mirax format, JPEG, TIFF
Pannoramic Scan®	3DHistech http://www.3dhistech.com/	20x/0.80 (0.23 μm) Plan Apo. 40x/0.95 Plan Apo	150	Fluorescence available	Mirax format, JPEG, TIFF
Pannoramic 250®	3DHistech http://www.3dhistech.com/	20x/0.80 (0.23 μm) Plan Apo. 40x/0.95 Plan Apo	250	Fluorescence available	Mirax format, JPEG, TIFF
NanoZoomer® HT	Hamamatsu http://www.hamamatsu.com/	20x/0.7. Modes: x20 (0.46 µm/pixel) x40 (0.23 µm/pixel)	210	Fluorescence available	JPEG, TIFF
NanoZoomer® RS	Hamamatsu http://www.hamamatsu.com/	20x/0.75. Modes: x10 (0.92 µm/pixel) x20 (0.46 µm/pixel) x40 (0.23 µm/pixel)	6	Fluorescence available	JPEG, TIFF
Nuance FX	CRi http://www.cri-inc.com/	Spectral range 420 – 720 nm	1	Bright field and fluorescence multispectral imaging	.im3 for multispectral; 24-bit TIFF for RGB
ScanScope® CS	Aperio http://www.aperio.com/	20x/0.75 Plan Apo	5	Bright-field, halogen lamp	TIFF (Aperio SVS), CWS
ScanScope® GL	Aperio http://www.aperio.com/	20x/0.75 Plan Apo	1	Bright-field, halogen lamp	TIFF (Aperio SVS), CWS
ScanScope® GL-E	Aperio http://www.aperio.com/	20x/0.75 Plan Apo	1	Bright-field, halogen lamp	Composite WebSlide (CWS)
ScanScope® OS	Aperio http://www.aperio.com/	60x/1.35 Plan Apo	1	Oil immersion	TIFF (Aperio SVS), CWS
ScanScope® XT	Aperio http://www.aperio.com/	20x/0.75 Plan Apo	120	Bright-field, halogen lamp	TIFF (Aperio SVS), CWS
Toco™	Claro http://www.claro-inc.jp/	Carl Zeiss Aplan 20x, 40x	1	Bright-field, flat LED	Claro format, JPEG
Vassalo™	Claro http://www.claro-inc.jp/	Carl Zeiss Aplan 20x,40x	80 or 20	Bright-field, flat LED	Claro format, JPEG

## COST Action IC0604

The COST Action IC0604 “Telepathology Network in Europe” (EURO-TELEPATH) is aimed to coordinate research efforts to develop the most adequate technological framework for the management of multimedia electronic healthcare records (data and images) applied to Anatomic Pathology. The integration of digital slides in the electronic health record is one of the main objectives of this COST Action.

Coordinated work in COST Action IC0604 is summarized in the following research directions:

1. **Automation procedures** in Pathology. Best technology available and under research.

2. Scanning solutions for Pathology microscopic slides.

3. Technological solutions for **compression and storage** problems with large image files.

4. Virtual slide standard viewer specifications which allow efficient reviewing of pathology images.

5. International standards (DICOM, HL7, SNOMED, CEN) and IHE initiative.

6. **Model for pathology** and other hospital information systems

7. An **European-scope telepathology** network

8. Collection of interesting and typical samples, and clinico-pathological sessions

## Digital slides in pathology

Current medical informatics standards are being adapted to manage efficiently extremely large medical images, like digital slide files.

In order to achieve digital slides integration in electronic health record, we need a multidisplinary work, including collaboration between informaticians, engineers, pathologists, technicians, clinicians, primary care professionals, and administrators. This means that we need to think globally, searching for solutions not only for specific departmental aims but also with a scope comprising hospital, regional, national, European, and international levels.

Digital slides can play a role in disease prevention (e.g. cervical cancer screening) [1]. In pathology slide reading, automated image analysis can also a valuable tool in the selection of areas of interest in conventional staining (HE, Papanicolaou), special techniques (Zielh-Neelsen, silver stains), immunohistochemistry, and in situ hybridization.

An automated screening assistance method has been successfully tested in tissue Ziehl-Neelsen-stained sections for acid-alcohol-fast bacilli detection [2].

In immunohistochemistry, quantitative evaluation of protein levels can be now assessed by applying digital image analysis. The importance of these studies is, amongst other, the capability to select the best treatment for each patient. The targeted therapeutic strategy requires both histologic and genetic data. In many cases, this quantitative evaluation allows a correlation between protein overall expression and the number of copies of a gene. Using these techniques in oestrogen receptor analysis, it has been found that although ESR-1 gene over-expression happens frequently in breast cancer, only a subset of these are high amplified cases correlated to increased response rates in hormonal therapy (tamoxifen) [3].

Digital image analysis can be applied not only to protein expression levels evaluation (e.g. Nuclear Labelling Index in p53) by immunohistochemistry but also in chromogenic in situ hybridization (CISH) and silver in situ hybridization (SISH) procedures (e.g. chromosome 17 in P53 and HER-2 genes) [4].

The advantage of CISH is that we can use the same bight field scanning system for conventional stains, immunohistochemistry and CISH or SISH. CISH has shown high concordance with fluorescent in situ hybridization (FISH) and has the potential to replace both the immunohistochemistry and FISH techniques for primary HER2 testing and eliminate the need for a triage system that requires 2+ immunohistochemistry results to be retested by FISH [5].

In FISH, special equipment for fluorescence digital slide scanning is needed. However, since skin and kidney biopsies and many in situ hybridization probes need the procedures using fluorescent techniques (FISH), scanning systems with fluorescence capabilities are needed in those departments with a high volume of skin and kidney biopsies or FISH studies.

In case that an image analysis system is used to score slides, the scanning system and the automated image analysis software must be standardized and validated against another method of measurement (for instance, in oestrogen/progesterone receptor studies) [5].

When multiple signals need to be analyzed simultaneously, multispectral imaging is a good solution. There are commercially available multispectral fluorescence- and brightfield-capable microscope slide analysis systems, that are able to scan cytology, tissue sections or tissue microarrays, stained with standard stains (H&E or trichrome stain), or immunofluorescence and immunohistochemical techniques.

In the near future, quantum dot-based immunofluorescent and other brigh tfield microscopy nanotechnology, combined with high resolution scanning will allow a better morphological observation and quantitative imaging of protein expression.

## Interoperability in pathology information systems

One of the first steps to achieve interoperability between different pathology information systems is obtaining a seamless integration of pathology images (gross pictures and digital slides) with LIS-Pathology Information system in a web environment.

Primary care centres play an essential role in the generation of clinical information and specimen collection, mainly cytology specimens, but the number and complexity of biopsies from primary care centres is increasingly growing.

A common terminology, based in SNOMED CT is also needed. Most countries are just beginning to be aware of the importance of terminology and the work of organizations like International Health Terminology Standards Development Organisation (IHTSDO) This means that most companies have little experience in SNOMED CT implementation beyond report coding. Additional problems are that many pathology departments are still using old versions of SNOMED (SNOMED II) and they are using many local codes. The general recommendation is that old SNOMED II codes should be converted into SNOMED CT codes. In many cases, code migration of the pathology reports from SNOMED II to SNOMED CT may be necessary.

**Figure 1:**
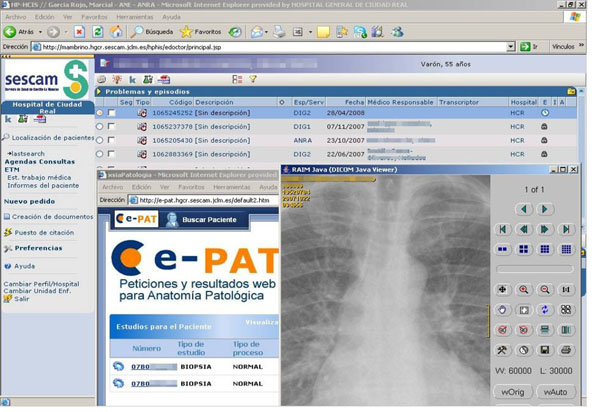
Hospital Information Systems (HP-HCIS), Pathology information system (e-PAT) and DICOM image viewer (RAIM Java) integration.

The work to be done in integration of pathology information systems (fig 1) can be described at user, patient, semantic, and knowledge levels. At user level, although user identification allows access to multiple applications, security needs to be improved since digital signatures are not widely used in these systems. At patient level, current solutions allow working with the same patient in different applications, and working with the same episode or visit; however, problems oriented integration is not achieved, and very few systems are able to classify reports or images according to problems or diagnoses. Primary care information systems should also be included in the integration. At the semantic level very little is achieved, and pathology information systems should be able to use efficiently ontologies, including relationships between terms in searches, and selection of appropriate hierarchies according to context. Finally, efficient knowledge management is possible if all information collected by pathology information systems is elaborated not only to improve quality (cyto-histologic correlation, frozen section studies accuracy), but also to offer a clear view of the work performed in pathology department.

## Conclusions

Current barriers in the integration of digital slides in eHealth record can be broken down. Cost of current digital slide scanners is one of the main barriers, and new financing solutions, like leasing or cost agreement per digitized slide are possible.

Pathology information system is the core where all others information system (imaging, laboratory workflow) must converge. For that reason it is very important that pathology information system vendors participate in standardization bodies (imaging, messaging, terminology), with the participation of pathologist and clinicians, including primary care doctors.

## Competing interests

The authors declare that they have no competing interests.
